# Health-related quality of life among Swedish children with Juvenile Idiopathic Arthritis: parent–child discrepancies, gender differences and comparison with a European cohort

**DOI:** 10.1186/s12969-017-0153-5

**Published:** 2017-04-12

**Authors:** Veronica Lundberg, Catharina Eriksson

**Affiliations:** Department of Clinical Sciences, Pediatrics, Umeå University, SE 901 85 Umeå, Sweden

**Keywords:** Gender, Informant discrepancies, Paediatrics, Questionnaire

## Abstract

**Background:**

This study investigates gender differences in self-reports and between parent and child reports in Health-related Quality of Life (HRQOL), measured with disease-specific and generic instruments for chronic disease. Comparison of HRQOL results in this Juvenile Idiopathic Arthritis (JIA) sample to a European cohort of children with JIA and one of children with other health conditions are also made.

**Methods:**

Fifty-three children with juvenile idiopathic arthritis (JIA), aged 8–18 years, and their parents completed the condition-specific DISABKIDS for JIA, and the DISABKIDS generic instrument for chronic conditions (DCGM-37) in a cross-sectional study. European reference data were used for comparison of child and parental reports.

**Results:**

Child self-reports in DCGM-37 and DISABKIDS for JIA showed no gender differences. Parental and child reports of the child’s HRQOL differed only in DCGM-37; this was among girls who scored their independence (*p* = 0.03), physical limitation (*p* = 0.01), social exclusion (*p* = 0.03), emotions (*p* <0.01), and general transformed score (*p* <0.01) higher than did their parents. Our sample of children with JIA reported more physical limitation compared to samples of European children with JIA (*p* = 0.01), European children with chronic conditions (*p* < 0.01), and their parents (*p* = 0.01 and *p* < 0.01). The Swedish children reported more problem with understanding compared to the European JIA sample (*p* = 0.03). Swedish parents perceived their children’s independence significantly lower than did the European parents of JIA children (*p* < 0.01), as well as European parents of children with chronic conditions (*p* = 0.03). The Swedish parents also perceived their children to have significantly lower social inclusion (p < 0.05) and general transformed score (p = 0.04), in comparison to European parents of children with chronic conditions.

**Conclusions:**

Parent–child differences in assessment of quality of life depend on the HRQOL instrument used, especially among girls. In comparison to European cohorts, our sample of children with JIA experienced more physical limitations and less understanding.

## Background

Juvenile idiopathic arthritis (JIA) encompasses all forms of arthritis that begin before the age of 16 years, persists for more than 6 weeks, and are of unknown cause [1–3]. JIA is classified into seven disease subtypes by to the International League of Associations for Rheumatology (ILAR) [4]. Improvement in functional outcomes has been documented, and is due to progress in disease management [1]. Still, growing up with JIA can be associated with risk for numerous functional limitations, e.g., swollen joints, medication side effects, pain, fatigue [3, 5, 6], and visual impairment [3, 6]. Children with JIA are more likely to report a lower health related quality of life (HRQOL), compared to normative data [2, 3, 7].

HRQOL measures can be divided into condition-specific measures, generic, or generic instruments for chronic conditions. Condition-specific instruments address aspects related to a specific condition, its treatment, or symptoms. Generic instruments are suitable to measure HRQOL across both healthy populations and populations with medical health conditions, while generic instruments for chronic conditions assesses HRQOL only among children with various chronic health conditions. In our previous study, we used the generic HRQOL instrument Pediatric Quality of Life inventory (PedsQL) in the same sample as in this present article, and found that more than half of girls and boys with JIA experienced suboptimal HRQOL compared to a healthy population [7].

Differences between self-report and parent-report (proxy-rater) has been demonstrated in HRQOL measurements of children with and without health conditions [8, 9]. However, there are mixed findings in earlier studies that consider gender differences in parental versus child reports. They suggest that a child’s gender may not be related to informant discrepancies, except in specific populations [10]. Our previous study showed gender differences between parent and child self-reports for the child HRQOL in JIA children, measured with the generic PedsQL. The most significant differences were among girls, who rated their HRQOL better in most subdomains compared to their parents [7].

To our knowledge, there have been no studies that evaluate parent–child discrepancies among different types of HRQOL instruments, e.g., disease-specific and generic HRQOL instruments for chronic conditions.

### Objectives

The primary aims of the present study were to evaluate child self-reported and parent-reported HRQOL with instruments for chronic conditions, and a condition-specific HRQOL instrument for JIA. We specifically wanted to evaluate for gender differences in HRQOL self-report, and to explore gender differences in parent report of a child’s HRQOL. The secondary aims were to compare the HRQOL results in this JIA sample to a European cohort of children with JIA and one of children with other health conditions.

## Methods

### Study design and study population

This cross-sectional questionnaire survey included children and adolescents registered with a diagnosis of JIA at the Department of Child and Adolescent Medicine, Norrlands University Hospital, Umeå, Sweden. The study population included all eligible children, identified through clinical appointment schedules at routine outpatient appointments, between September 2009 and December 2010.

### Participants

Eligibility criteria were children aged 8 to 18 years with a JIA diagnosis defined by the ILAR classification criteria [4]. Of 69 eligible children, 56 children and their parents agreed to participate. The reason for declining participation was unknown in six families, four parents said that their child only had small problems due to JIA, two cited a lack of time, and one child was discharged from the clinic. Three were excluded because of a missing parental report. Therefore, 53 children (38 girls and 15 boys) remained as the study group. Of the participating parents, 37 were mothers, 12 were fathers, and in four families the mothers and fathers completed the questionnaires together. For nine of the girls, at least one additional diagnosis was reported by the child or parent, i.e., uveitis, overweight, attention-deficit/hyperactivity disorder, asthma, dermatitis, Turners syndrome, celiac disease, Crohn’s disease, or thyroid gland disease. Among five of the boys, at least one additional diagnosis was reported, i.e., asthma, allergy, panic disorder, Raynaud’s syndrome, or lactose intolerance.

### Measures

#### HRQOL

The DISABKIDS generic instrument for chronic conditions (DCGM-37) consists of 37 items assigned to six sub-scales: *independence*, *emotional*, *social inclusion*, *social exclusion*, *physical limitation*, and *medication*. DCGM-37 was tested in 2002–2003 cross-culturally in seven European countries, including Sweden. The findings with respect to internal consistency (Chronbachs’Alpha: 0.70–0.87 in child version and 0.77–0.90 in proxy version) and test-retest reliability (0.71–0.83) has been demonstrated. The results indicate that DCGM-37 is suitable for HRQOL comparisons in children and adolescents with different diseases and across cultures [11–13]. The condition-specific DISABKIDS for JIA addresses arthritis-specific symptoms and limitations. Its 12 items are assigned to two sub-scales: *impact*, and *understanding*. The internal consistency of each sub-scale was between 0.73 and 0.87, in a field study among children with JIA [13]. Both the JIA DISABKIDS and DCGM-37 are suitable for use between ages 8 and 16 years. Each item relates to symptoms during the last four weeks, and was answered on a five-point Likert scale between 1 (never) to 5 (always). In our sample, when a child or parent registered an odd number, the score was rounded to the inferior option. The scale scores were transformed with the syntaxes for DISABKIDS scales, to a range of 0 to 100, with higher values indicating better HRQOL [12]. Both self-report and an equivalent parent version was included.

*European reference data*, obtained from the DISABKIDS manual [12] were used for comparison with the Swedish sample of JIA patients. The reference data, based on a field study, included a European cohort of *n* = 148 children with JIA, and a cohort of *n* = 1152 participants with the following chronic conditions; arthritis, asthma, atopic dermatitis, cerebral palsy, cystic fibrosis, diabetes and epilepsy [12].

*Disability* was measured by the Childhood Health Assessment Questionnaire (CHAQ) [14], composed of 30 items that measure eight functional areas. Each question was scored on a four-point scale between 0 (no difficulty) and 3 (unable to do). The question with the highest score determined the score for that functional area. The scores for each of the eight functional areas were averaged to calculate a Disability Index (DI) that ranged from 0 (no disability) to 3 (disabled) [14]. Suggested cut-off levels for disability are at medians of 0.13 (mild), 0.63 (mild-to-moderate), and 1.75 (moderate) [15]. The original CHAQ showed internal reliability (Chronbachs’Alpha 0.94) and test-retest reliability with Spearman’s correlation coefficient of 0.8 and convergent validity between Kendall’s tau; 0.54–.77. The Swedish version of CHAQ showed internal reliability of Cronbach’s alpha 0.89, test-retest reliability for the disability index was high (r(S) =0.92, r(P) = 0.97; *p* <0.00001) [14, 16].

*Disease duration* in years was calculated from medical record data, based on the date of diagnosis and according to ILAR classification criteria.

*Disease activity* was measured by a paediatric rheumatologist who assessed global disease activity into categories of no, mild, moderate, or high. Mild disease activity was defined as between no activity and moderate activity, moderate activity meant the child needed changes in medical therapy, and high activity captured severe immunologic and clinical activity. Also erythrocyte sedimentation rate (ESR) in mm/hour was used.

*Medications* were compiled into four categories: 1) disease modifying anti-rheumatic drugs (DMARDs), e.g., azathioprine, cyclosporine, hydroxychloroquine, methotrexate, and sulfasalazine; 2) non-steroidal anti-inflammatory drugs (NSAIDs); 3) biological medications, e.g., adalimumab, anakinra, etanercept, infliximab and rituximab; and 4) corticosteroid therapy (Table 1).

**Table 1:** Characteristics of 53 children with juvenile idiopathic arthritis aged 8–18 years

	Girls (*n* = 38)	Boys (*n* = 15)	*P*-value^a^
**Age** in years, Md (range)	13 (8–18)	15 (10–18)	0.51
8–12 years, *n* (%)	16 (42)	5 (33)	
13–18 years, *n* (%)	22 (58)	10 (67)	
**CHAQ Disability Index** ^b^, Md (range)	0.50 (0–2.75)	0.38 (0–1.38)	0.65
Categories, *n* (%)			0.56
None-mild	12 (32)	5 (33)	
Mild-moderate	9 (24)	5 (33)	
Moderate	13 (34)	5 (33)	
Severe	3 (8)		
Missing	1 (3)		
**Pain VAS** ^c^, 0–100 Md (range)	32 (0–93)	50 (0–99)	0.50
**Well-being VAS** ^d^, 0–100 Md (range)	22 (0–99)	27 (0–100)	0.75
**ILAR diagnosis** ^e^, ***n*** (**%)**			*0.01*
Systemic arthritis	1 (3)	1 (7)	
Oligoarthritis	16 (42)	3 (20)	
Polyarticular arthritis, RF^f^-negative	2 (5)		
Polyarticular arthritis, RF^f^-positive	8 (21)	1 (7)	
Psoriatic arthritis	6 (16)		
Enthesitis-related arthritis	3 (8)	5 (33)	
Unspecified arthritis	2 (5)	5 (33)	
**Physical education participation**,			
*n* (%)			0.08
Always	12 (32)	9 (60)	
Sometimes	16 (42)	4 (27)	
Not at all	6 (16)	2 (13)	
Not relevant	3 (8)		
Missing	1 (3)		
**Disease duration** in years Md (range)	4.5 (0–16)	3.0 (0–13)	0.09
**ESR** ^g^, mm/hour, Md (range)	5 (2–31)	4 (2–35)	0.14
**Disease activity categories**, *n* (%)			0.37
No	13 (34)	8 (53)	
Low	20 (53)	5 (33)	
Moderate	4 (10)	2 (13)	
High	0	0	
Missing	1 (3)	0	
**Medication at study visit** ^h^, *n* (%)			
DMARD	24 (63)	7 (47)	0.70
NSAID	25 (66)	8 (53)	0.42
Biologic medication	4 (10)	0	
Corticosteroid therapy	7 (18)	0	
Eye drops/corticosteroid	2 (5)	0	

^a^Mann Whitney *U* test

^b^Childhood Health Assessment Questionnaire Disability Index

^c^Visual Analogue Scale for pain during the past week; 0 = no pain and 100 = very severe pain

^d^Visual Analogue Scale for assessment of overall well-being; 0 = very good and 100 = very bad

^e^JIA- diagnosis defined by the International League of Associations for Rheumatology (ILAR) classification criteria

^f^Rheumatoid factor

^g^Erythrocyte sedimentation rate

^h^Disease modifying anti-rheumatic drug (DMARD), Non-steroid anti-inflammatory drug (NSAID)

### Procedures

Families who agreed to participate in the study completed a mental health questionnaire; the Strengths and Difficulties Questionnaire and three HRQOL questionnaires, of which the DISABKIDS for JIA and the DCGM-37 were included in this study. The third HRQOL questionnaire, PedsQL is presented in an earlier article [7]. All these questionnaires were answered prior to a scheduled medical visit at the paediatric outpatient clinic. Parents and children completed the questionnaires separately, and a research assistant was available for the child. The CHAQ is used routinely in clinical practice, and was answered by children and their parents at home before the outpatient clinic visit. Estimated time to complete all of the study questionnaires was 20–40 min for each child, plus another ten minutes for the CHAQ to be completed at home. Nor the children or parents were reimbursed for their time participating in this study.

### Statistics

Data from girls, boys, and their parents were analysed separately and as one group. Results of the descriptive analysis are presented as median and range, or numbers (n) and percentages (%). HRQOL results are presented as mean, standard deviation (SD), median (Md), and range. Mann Whitney *U* test was used to analyse gender differences in characteristics and to compare the median of HRQOL between boys and girls. The Wilcoxon signed-rank test was used to analyse differences between self-reports and parental reports of HRQOL. Student’s one sample *t*-test with hypothesized mean values obtained from the European reference data in tables 4–11, 4–12, 4–15, 4–16 and 4–278 in the DISABKIDS manual were used to evaluate for HRQOL differences between the Swedish JIA sample and European samples [12]. The analyses were performed using Statistical Package for the Social Sciences (SPSS) version 18, 19 and 22. Statistical significance was defined as *P* **<** 0.05.

## Results

### Sample characteristics

The 53 children with JIA had a median age of 14 years (range 8–18). The paediatric rheumatologist categorized no or low disease activity among 87% of the girls and 86% of the boys. Girls and boys differed significantly only in JIA sub-diagnosis (Table 1).

### Generic HRQOL for chronic disease

There were gender differences between child self-report and parental report in DCGM-37. The girls and their parents differed significantly in the subscales of *independence*, *physical limitation*, *social exclusion*, *emotion*, and *general transformed score*. Among the boys, there were no differences between self-reports and parental reports on any subscales or for the total scale score. There were no differences between girls’ and boys’ self-report for any of the six DCGM-37 subscales, total general transformed score, or the 37 specific questions included in the questionnaire. However, among the parents’ answers in the DCGM-37 *social exclusion* dimension, parents of boys reported higher (better) child scores than did parents of girls (*P* = 0.05) (Table 2).

**Table 2:** Health related quality of life measured with DISABKIDS generic instrument for chronic conditions (DCGM-37), and differences in self-report and parent-report

	Girls *n* = 38 Mean (SD)	Girls’ parents *n* = 38 Mean (SD)		Boys *n* = 15 Mean (SD)	Boys’ parents *n* = 15 Mean (SD)		Girls parents versus boys parents	Girls versus boys
	Median (range)	Median (range)	*P*-value^1^	Median (range)	Median (range)	*P*- Value^1^	*P*- Value^2^	*P*- Value^3^
Independence	75.66 (13.55)	69.71 (17.51)		71.11 (21.10)	75.28 (19.51)		0.23	0.67
	75.00 (50.00–100)	70.83 (29.17–100)	*0.03*	75.00 (25.00–100)	79.17 (20.83–100)	0.17		
Physical limitation	65.94 (21.04)	58.66 (22.25)	*0.01*	62.78 (24.42)	66.67 (24.14)	0.21	0.11	0.70
	66.67 (20.83–100)	54.17 (20.83–100)		62.50 (8.33–100)	70.83 (0.00–100)			
Social inclusion	70.94 (17.82)	66.99 (18.82)	0.18	69.72 (23.06)	72.94 (19.55)	0.67	0.19	0.92
	75.00 (37.50–100)	62.50 (37.50–100)		66.67 (20.83–100)	75.00 (20.83–100)			
Social exclusion	83.55 (15.59)	78.24 (19.11)	*0.03*	83.61 (17.64)	87.22 (21.45)	0.26	*0.05*	0.85
	87.50 (33.33–100)	81.25 (33.33–100)		87.50 (33.33–100)	91.67 (16.67–100)			
Emotion	75.99 (23.48)	67.09 (23.08)	*<0.01*	70.00 (22.66)	71.30 (26.02)	0.92	0.48	0.32
	82.14 (7.14–100)	66.07 (10.71–100)		67.86 (14.29–100)	78.57 (7.14–100)			
Medication	71.59 (25.67)	65.62 (2.93)	0.11	84.09 (15.68)	75.32 (18.28)	0.11	0.36	0.19
	77.08 (20.00–100)	75.00 (0.00–100)		87.50 (54.17–100)	79.17 (50.00–100)			
Missing^a^	6	2		4	2			
General transformed score	74.24 (16.13)	67.93 (17.59)	*<0.01*	73.31 (17.99)	74.89 (18.60)	0.90	0.16	0.94
	78.77 (37.50–100)	69.93 (30.41–96.8)		73.65 (29.05–100)	74.19 (23.65–100)			

^a^Missing referring medication

^1^Wilcoxon signed rank test. *P*-value between child and parent report

^2^Mann Whitney *U* test. *P*-value between parents of girls and parents of boys, with JIA

^3^Mann Whitney *U* test. *P*-value between girls and boys with JIA

### JIA-specific HRQOL

Children reported a mean condition-specific *impact* score of 60.30 (SD 22.21) and an *understanding* mean score of 60.58 (SD 24.15). The parental mean *impact* score was 57.98 (SD 21.34); the *understanding* mean score was 63.83 (SD 25.79). No significant differences between genders were found in the self-report for condition-specific DISABKIDS for JIA questionnaire, or between the children and their parents.

### Comparison with the European reference data cohort

On the DCGM-37, the Swedish JIA children scored significantly lower on the *physical limitation* subscale than both the European JIA children (*P* = 0.01) or European children with chronic conditions (*P* < 0.01). The parents reported significantly lower scores on child *physical limitation* compared to European parents of JIA children (*P* = 0.01) or European parents of children with chronic conditions (*P* < 0.01). The Swedish parents perceived their children’s *independence* to be significantly lower than the European parents of children with JIA (*P* < 0.01), as well as the European parents of children with a chronic condition (*P* = 0.03). In comparison to the Swedish sample, European parents of children with chronic conditions perceived their children to have significantly better *social inclusion* (*P* < 0.05) and general transformed score (*P* = 0.04) (Table 3).

**Table 3:** DISABKIDS generic instrument for chronic conditions (DCGM-37) in Swedish children aged 8–18 years, parents and a European sample aged 8–16 years

	Swedish Sample of JIA children *n* = 53 Mean (SD)	European sample of JIA children^1^ *n* = 148 Mean	*P*-value^2^	European sample of children with chronic conditions^3^ *n* = 1.152 Mean (SD)	*P*-value^4^	Swedish sample of parents of children with JIA *n* = 53 Mean (SD)	European sample of parents of children with JIA^5^ *n* = 141 Mean	*P*-value^6^	European sample of parents of children with chronic conditions^7^ *n* = 1.152 Mean (SD)	*P*-value^8^
Independ-ence	74.37 (15.96)	77.58	0.10	76.90 (18.34)	0.24	71.29 (18.09)	79.34	*<0.01*	76.60 (17.25)	*0.03*
Physical limitation	65.05 (21.85)	73.02	*0.01*	73.85 (18.23)	*<0.01*	60.92 (22.85)	68.54	*0.01*	70.19 (18.32)	*<0.01*
Social inclusion	70.60 (19.22)	72.86	0.37	75.25 (17.81)	0.10	68.68 (19.03)	72.59	0.10	74.28 (17.65)	*<0.05*
Social exclusion	83.57 (16.02)	85.79	0.27	85.15 (15.56)	0.52	80.78 (20.01)	82.69	0.42	80.91 (16.79)	0.94
Emotion	74.29 (23.19)	73.02	0.69	76.72 (20.56)	0.40	68.28 (23.78)	70.24	0.60	71.61 (20.49)	0.26
Medication	74.79 (23.98)	67.98	0.07	72.25 (22.71)	0.45	68.20 (25.12)	66.44	0.54	69.90 (22.15)	0.62
Missing^9^	10					4				
General transformed score	73.98 (16.51)	76.13	0.38	76.99 (14.22)	0.19	69.90 (17.98)	74.12	0.10	74.90 (14.55)	*0.04*

^1^European field study sample of children with arthritis (12) table 4–11

^2^One sample *t*-test. *P*-value between samples of Swedish JIA children and European JIA children

^3^European field study sample of children with chronic conditions: asthma, arthritis, dermatitis, diabetes, cerebral palsy, cystic fibrosis, epilepsy (12), table 4–15

^4^One sample *t*-test. *P*-value between Swedish sample of children with JIA and European sample of children with chronic conditions

^5^European field study sample of parents of children with JIA (12) table 4–12

^6^One sample *t*-test. *P*-value between Swedish sample of parents of children with JIA and European sample of parents of children with JIA

^7^European field study sample of parents of children with chronic conditions (12), table 4–16

^8^One sample *t*-test. *P*-value between Swedish parents of children with JIA and European parents of children with chronic conditions

^9^Missing referring medication

In the JIA-specific HRQOL instrument, our Swedish JIA children rated the *understanding* scale significantly lower than the European JIA sample: 60.58 (SD 24.15) versus 67.75 (SD 26.73) (*P* = 0.03).

## Discussion

We found no differences between parental and child reports with the condition-specific HRQOL instrument DISABKIDS for JIA. Nor did we find a gender difference in children’s self-reports. However, there were gender differences between parental and child reporting in DCGM-37; girls reported significantly better scores on several HRQOL sub-scales compared with parental reports. This confirms our previous finding in this same sample when the generic instrument PedsQL was used [7]. In the generic questionnaire PedsQL, the parent–child differences were also evident among the girls, who tended to reported better physical health, psychosocial health, and total HRQOL compared to their parents’ reports [7]. Also in accordance with our previous results [7], the boys tended to score lower on several HRQOL sub-scales, in both the conditions-specific DISABKIDS for JIA and the DCGM-37, compared to their parents. Such a scoring difference was not significant in the present study. Some authors found gender differences, with lower parent–child agreement in mental health between girls and their parents [10, 17], while others found inconsistent results [10]. Differences in agreement that depend on whether the mother or father is the proxy-rater were not explored in this study. A review by Upton et al. found a possible interaction of age and gender in parent–child agreement on HRQOL ratings; this depended on the gender of the parental proxy-rater. Fathers and mothers can have different perspectives on child health and behaviour, and sons and daughters might also differ in their relationship with each parents [8]. Mothers’ and fathers’ ratings correlate moderately, and bigger differences are seen between father/child ratings than for mother/child ratings [9].

Eiser et al. describe in their review, that a parent’s own well-being may affect the report of their child’s HRQOL and symptoms. If the parents report higher levels of emotional distress, they also report more negative perceptions of their child’s HRQOL [9]. However, Haverman et al. found that the parents of children with JIA self-reported their own HRQOL similar to parents of healthy children [18]. Specific HRQOL subdomains are suggested to affect parent–child agreement, with better agreement in observable behaviours such as physical functioning than in internal symptoms such as pain, emotional distress, or fatigue [9]. For example, in the PedsQL the majority of items involve observable items and are suggested to reflect good agreement [8]. However, Upton et al. found divergent findings for parent–child agreement on specific subscales, e.g., observable characteristics such as physical health and social and emotional functioning in healthy children and children with different health conditions [8].

Child self-report may be more reliable for information regarding minor, short-term effects of treatment, moment-to-moment HRQOL and disease-specific symptoms, whereas parents report might reflects overall symptoms occurring over time [9, 19]. Children might also report more symptoms, but less impact of their perceived difficulties than do their parents [19]. In children with a diagnosed medical condition, parental involvement and understanding increases regarding their child’s health issues and well-being [8, 9]. Even level of disease activity seems to affect parent–child agreement among children with polyarticular JIA. If the child had active disease, the parent–child agreement was higher than among children with inactive disease [20]. Parents of children with JIA might perceive their children as vulnerable, especially when the children have a shorter disease duration, higher degree of functional disability, or greater disease activity [18]. On the other hand, our sample of children did not differ in any of these characteristics. This view would therefore not explain the significant inter-rater differences on the DCGM-37 among girls. ILAR diagnosis was the only variable that differed among girls and boys in our sample. Parents are not in position to observe their children in environments outside the family, or how these influence the child’s feelings and function on a daily basis. And this is especially true for school-aged children and adolescents [9]. Inter-rater agreement can depend on the developmental level and age of the child, as well as the characteristics of the illness [9]. Yet, the authors found divergent results in a review that compared parent–child agreement in relation to child’s age. This ranged from no correlation with the child’s age to lower concordance between parents and adolescent children [8]. Due to the small sample, we did not evaluate for age differences in our study.

The sub-scales in the condition-specific DISABKIDS for JIA are supposed to be sensitive to treatment and improvement of the condition. The subscales *impact* and *understanding* are related to the invisibility and fluctuation of JIA symptoms [12]. In comparison to the European sample of children with JIA, our Swedish sample rated their *understanding* subscale significantly lower. This implies greater feelings of social exclusion and lack of empathy by peers and teachers. This suggests that Swedish children with JIA need more interventions in health education within school-based services [12].

The Swedish children with JIA and their parents scored significantly lower on the child *physical limitation* DCGM-37 subscale compared to the European children with JIA, European children with chronic conditions, and their parents. This indicates that our sample of children with JIA experience more functional limitations that are caused by JIA, and might need targeting intervention regarding exercise training [12]. Children with JIA have reduced anaerobic capacity and muscle strength. This results in physical, mental, and metabolic disorders. Exercise training is beneficial for JIA children, and leads to improved muscle strength, range of motion, quality of life, and reduced joint stiffness, without affecting disease activity [21]. Therefore, preventing physical limitations and supporting exercise training is essential in JIA. The Swedish parents perceive their children’s *independence* to be significantly lower compared to European parents of children with JIA, and European parents of children with a chronic condition. Compared to Swedish parents, European parents of children with chronic conditions perceive their children to have better *social inclusion* and a higher general transformed score. Comparison between these samples should be interpreted with caution since the characteristics and eventual comorbidity of the European samples are not described in detail [12]. A selection bias might therefore be evident, since the participants in the Swedish sample might not be exchangeable to those in the European cohort. The clinical relevance of different HRQOL domains may also vary between subgroups within broader disease groups like JIA. This makes meaningful comparisons difficult [8]. We do not know what demands our sample requires in daily life, or if these requirements differ between European countries; e.g., physical demands in school physical education classes, sports or recreational activities.

Possible bias in the results could be present because DISABKIDS assesses children 8–16 years of age, and we included children 8–18 years of age. The reason for including even 16–18 year old participants for us, were to evaluate for differences in parent–child discrepancies between three different HRQOL instruments; this present study and our previous study with the same sample [7]. Also, in our sample there was a relatively high frequency of comorbidity related to JIA or unrelated conditions. These may have influenced our results. Four children declined participation because they only had minor symptoms of JIA. This might have biased our results towards a lower HRQOL in some subscales. This sample size was small, especially among the boys. Only 15 boys were available for the study, and the comparisons involving the boys might therefore be underpowered. The multiple comparisons might also have resulted in significant results by chance. Other possible confounders such as socioeconomic status or parental education were not measured in this study.

Extensive attention has thus been given to examining informant characteristics that affect parent–child differences. Our results in the present and an earlier study show that the HRQOL instrument used can also influence parent–child differences in HRQOL. Differences, especially among girls, were evident in generic HRQOL measurement in our previous study [7], and in the DISABKIDS generic HRQOL instrument for chronic conditions in this study. But no differences were evident in the conditions specific HRQOL instrument DISABKIDS for JIA. To our knowledge this is the first study investigating parent–child proxy-rate discrepancies in different HRQOL instruments. Inconsistent or no differences exist for many of the child and parent characteristics reviewed in earlier studies (e.g., child’s age, gender, problem type, HRQOL subdomains, social desirability, child and parent health, relationship) with regard to parent–child differences in reports of the child’s HRQOL. Knowledge of all available research is essential to take into account when evaluating child self-report of HRQOL. Integrating and analysing all such variables into paediatric healthcare might be impractical. However, implementation of routine HRQOL measurements in clinical care is an important goal for assessment of paediatric patients with chronic health conditions like JIA. This is especially the case since JIA involves fluctuating disease-activity, long-term treatment, common medication side effects, social and physical limitations, and comorbidities related or unrelated to JIA. Facilitating child self-report is essential when one considers the many variables that can affect parent–child differences in HRQOL measurement. Child self-report should be complemented by parent-report in order to give a more complete report of the child’s health.

We agree with other authors [8, 9], that information from both the child and parent provides a richer picture and invaluable information. Therefore, both need to be included whenever evaluating a child’s HRQOL. If the child is unable to self-report, a condition specific HRQOL measurement like DISABKIDS for JIA, might be preferable for proxy-report and best reflect the child’s own opinion of their HRQOL at the time of evaluation. Routine HRQOL measurement in the clinic can promote targeted interventions from healthcare professionals. For example, interventions could be directed toward the physical limitations and understanding evident in our sample.

## Conclusions

Differences in parent vs. child self-report were evident among girls with JIA when measured with the generic instrument for chronic disease, DCGM-37. These results may indicate that parent–child differences depend on the type of HRQOL instrument used. There was no gender differences among children’s self-report on any of the HRQOL instruments used in this study. In comparison to a European cohort, Swedish children with JIA experience more physical limitation and problems with understanding.
